# Supplementary figures and images for: The parietal cortex has a causal role in ambiguity computations in humans
Source: PLoS Biol. 2024 Jan 10;22(1):e3002452. doi: 10.1371/journal.pbio.3002452 (PMC10824459; doi:10.1371/journal.pbio.3002452)

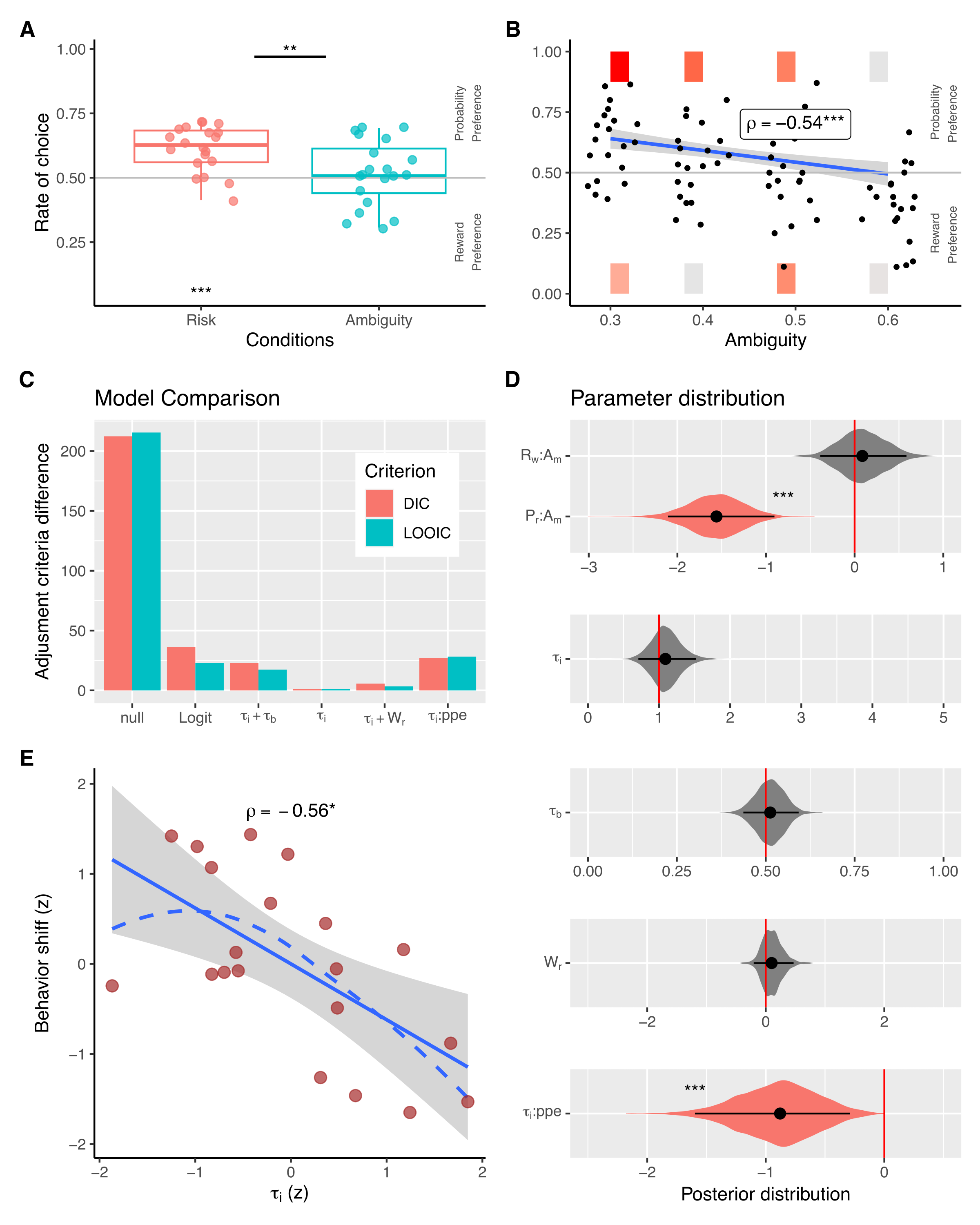

Supplement: S1 Fig — (A) Rate of choice where individuals preferred the highest probability per conditions. (B) Rate of choice where subjects preferred the highest probability per degree of ambiguity. Black dots represent the rate per individual. Color rectangles indicate the number of individual decisions; red represents the maximum, and light gray, represents the minimum account. The blue line represents the linear regression, and the gray area is the standard error. (C) Model fitting comparison using DIC, red, and LOOIC, green. (D) Posterior distribution of the key parameters for each model. Black dots represent the mean of the distribution, and black lines the 95% high-density intervals. The colored areas represent the complete posterior distribution. (E) Correlation between decision shift (difference between the rate of choices that subject prefers the highest probability between condition, Ambiguity less Non-ambiguity) and parameters. Red dots represent each subject. The solid blue line represents the linear regression, the dotted blue line the LOESS regression, and the gray area represents the standard error; * indicates p < 0.05, ** p < 0.01,***p < 0.001. The data underlying this figure can be found at https://osf.io/zd3g7/. (PNG) [file pbio.3002452.s001.png]

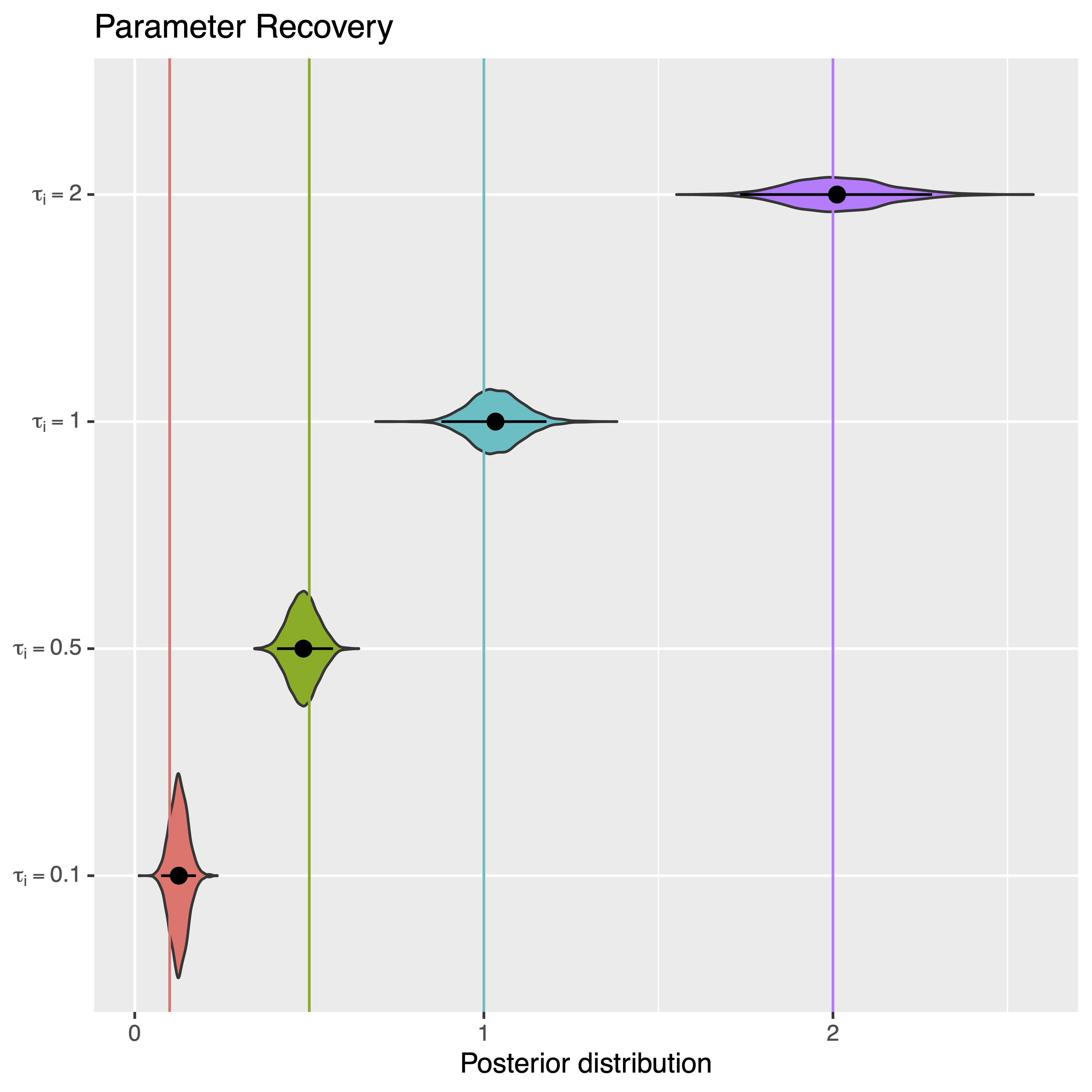

Supplement: S2 Fig — Posterior distribution of τi parameter recovery from simulated dated generated by different tau parameters. For the simulation, all the other model parameters were fixed using the mean of the posterior distribution fitted from the real data. The data underlying this figure can be found at https://osf.io/zd3g7/. (PNG) [file pbio.3002452.s002.png]

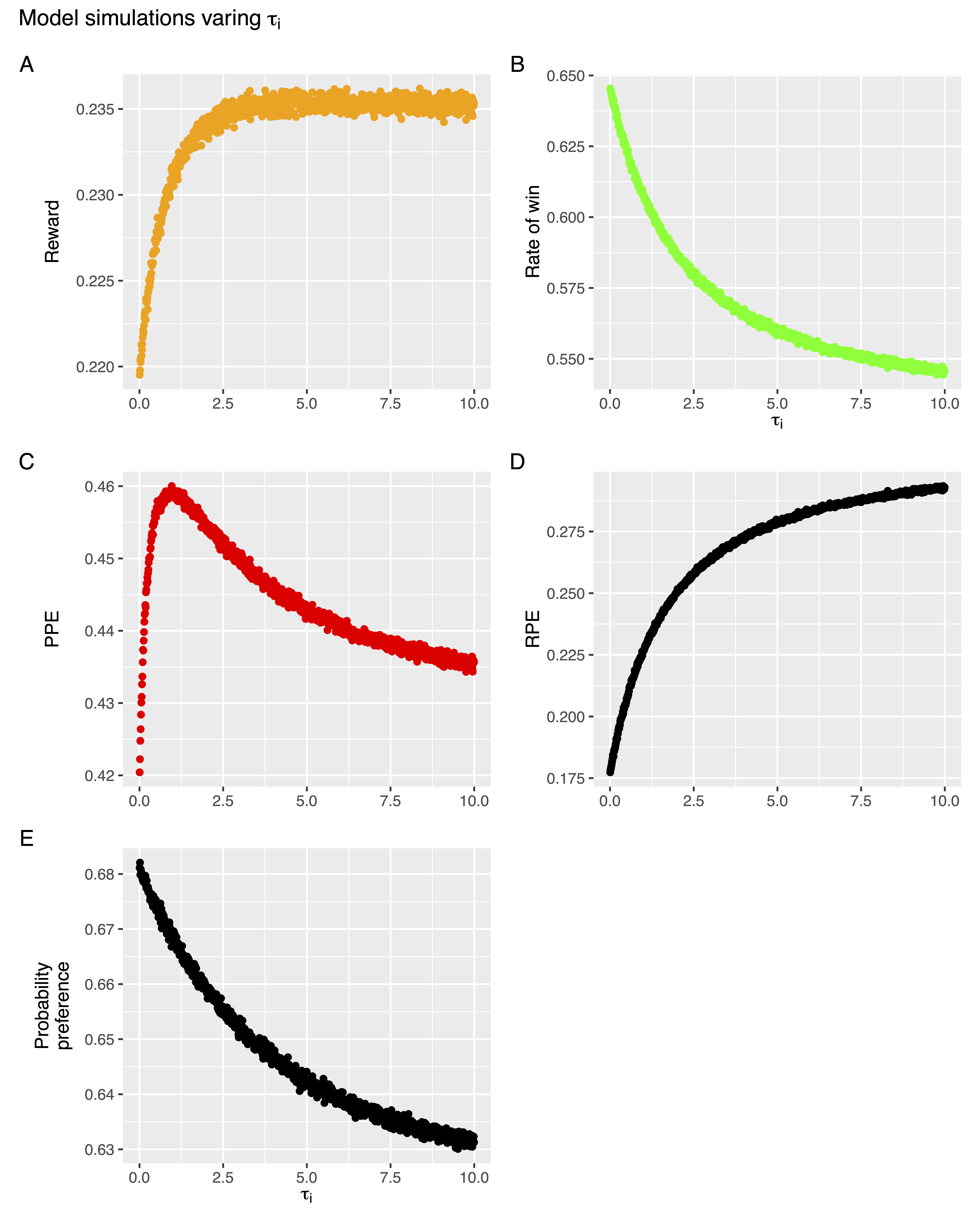

Supplement: S3 Fig — (A) Mean reward obtained in a game as a function of the τi parameter. (B) Mean rate of positive feedback or winning obtained in a game as a function of the τi parameter. (C) Mean probability prediction error (PPE) obtained in a game as a function of the τi parameter. (D) Mean reward prediction error (RPE) obtained in a game as a function of the τi parameter. (E) Mean rate of choosing the more probable option in a game as a function of the τi parameter. The data underlying this figure can be found at https://osf.io/zd3g7/. (PNG) [file pbio.3002452.s003.png]
