# Supplementary material for: The parietal cortex has a causal role in ambiguity computations in humans
Source: PLoS Biol. 2024 Jan 10;22(1):e3002452. doi: 10.1371/journal.pbio.3002452 (PMC10824459; doi:10.1371/journal.pbio.3002452)
Supplement: S1 Table — Parameters of the different behavioral model adjusted. The data underlying this table can be found at https://osf.io/zd3g7/. (PDF) [file pbio.3002452.s004.pdf]

Supplementary Table 1

fMRI behavioral Models

MODEL: Logit

	Lower95	Median	Upper95	Mean	SD	Mode	psrf
beta0	-3,531	-3,074	-2,583	-3,074	0,244	-3,055	1,074
beta1 PV	5,789	6,775	7,764	6,790	0,508	6,745	1,077
beta2 Rw	0,819	1,282	1,785	1,289	0,248	1,269	1,014
beta3 PV*AM	-4,637	-3,929	-3,126	-3,908	0,399	-3,947	1,081
beta4 Rw*AM	-0,396	-0,168	0,074	-0,166	0,123	-0,167	1,018
beta5 AM	1,445	1,855	2,231	1,844	0,204	1,900	1,080
deviance	1.408,430	1.437,135	1.470,610	1.437,823	15,727	1.437,550	1,009

MODEL: null (Prospect theory)

	Lower95	Median	Upper95	Mean	SD	Mode	psrf
alpha	1,102	1,602	2,056	1,610	0,245	1,614	1,012
gamma	1,802	2,522	3,374	2,549	0,408	2,485	1,006
beta_0	-0,043	0,088	0,215	0,086	0,068	0,085	1,003
beat_1	6,454	10,426	15,155	10,652	2,231	10,014	1,019
deviance	1.599,340	1.626,300	1.657,740	1.626,962	14,966	1.625,050	1,004

MODEL: tau_i

	Lower95	Median	Upper95	Mean	SD	Mode	psrf
tau_i	1,250	1,819	2,478	1,848	0,322	1,794	1,002
alpha	0,699	1,115	1,678	1,133	0,252	1,089	1,002
gamma	2,552	3,890	5,475	3,900	0,729	3,923	1,010
beta_0	0,012	0,150	0,282	0,148	0,068	0,145	1,006
beta_1	11,750	22,486	42,344	24,469	8,264	20,948	1,011
deviance	1.330,300	1.363,900	1.396,900	1.364,200	17,092	1.363,400	1,001

MODEL: tau_i + tau_b

	Lower95	Median	Upper95	Mean	SD	Mode	psrf
tau_b	0,468	0,507	0,547	0,507	0,021	0,507	1,001
tau_i	0,989	1,451	1,971	1,474	0,259	1,403	1,002
alpha	0,608	1,097	1,791	1,151	0,313	1,015	1,010
gamma	2,113	3,661	5,552	3,765	0,883	3,475	1,007
beta_0	-179,972	2,547	207,589	2,651	99,241	7,229	1,000
beat_1	10,422	20,533	55,917	24,283	12,639	18,495	1,021
deviance	1.322,880	1.358,205	1.394,250	1.358,304	18,387	1.358,770	1,000

MODEL: tau_i + Wr

	Lower95	Median	Upper95	Mean	SD	Mode	psrf
tau_i	1,247	1,853	2,614	1,893	0,348	1,791	1,006
alpha	0,800	0,957	1,162	0,970	0,108	0,889	1,001
Er	-0,122	0,488	1,287	0,531	0,362	0,445	1,011
Wr	0,165	2,539	4,254	2,425	1,140	2,850	1,001
beta_o	0,014	0,163	0,313	0,163	0,076	0,174	1,016
beta_1	13,031	20,098	29,514	20,816	4,691	19,407	1,007
deviance	1.325,420	1.357,860	1.392,880	1.358,047	17,281	1.357,207	1,002
