# Supplementary material for: The parietal cortex has a causal role in ambiguity computations in humans
Source: PLoS Biol. 2024 Jan 10;22(1):e3002452. doi: 10.1371/journal.pbio.3002452 (PMC10824459; doi:10.1371/journal.pbio.3002452)
Supplement: S2 Table — Significant cluster for the fMRI models. The data underlying this table can be found at https://osf.io/zd3g7/. (PDF) [file pbio.3002452.s005.pdf]

Supplementary Table 2

fMRI Decision Model 1

Regressor: Rw

Cluster	Voxels	P	-log10(P)	Z-MAX	Z-MAX X	Z-MAX Y	Z-MAX Z	Z-COG X	Z-COG Y	Z-COG Z	COPE-	COPE-	COPE-	COPE-	COPE-
19	2473	9,90E-25	24	5	2	20	50	-3,6	15	50,2	106	-32	-4	70	37,3
18	2225	5,45E-23	22,3	4,38	-44	-30	36	-37,8	-43,6	50,4	138	-48	-40	60	46,6
17	1012	3,04E-13	12,5	4,25	28	-54	-24	24,4	-65,2	-21,5	69,3	28	-76	-18	30,7
16	961	9,12E-13	12	5,28	16	18	10	14,1	6,95	5,24	53,7	8	6	4	23,2
15	949	1,19E-12	11,9	5,22	-12	6	0	-13,8	10,2	1,54	63,2	-6	2	10	26,2
14	358	3,40E-06	5,47	4,29	50	-70	2	51,1	-65,7	-2,31	49,2	52	-66	0	28,9
13	326	9,12E-06	5,04	4,03	-54	14	0	-51,7	9,56	22,4	67	-54	18	-4	35
12	315	1,29E-05	4,89	4,31	-42	54	12	-40,2	50,1	10	55,2	-42	54	12	33,6
11	304	1,82E-05	4,74	4,29	30	-38	44	38,6	-41,1	52	76,2	48	-38	62	41,4
10	299	2,15E-05	4,67	4,23	36	-2	52	32,9	-3,98	56,6	87,5	32	4	66	40,2
9	211	4,25E-04	3,37	4,15	-14	-18	4	-10,8	-18,8	7,3	31	-2	-12	10	15,8
8	207	4,92E-04	3,31	4,36	60	14	2	54,5	11	14,1	53,1	60	14	2	29,6
7	195	7,63E-04	3,12	3,97	12	-70	56	16,6	-68,9	52,9	82,2	12	-70	62	49,9
6	179	1,39E-03	2,86	4,37	-32	-68	-24	-32,9	-58,6	-26,3	36,8	-32	-68	-22	23,5
5	168	2,13E-03	2,67	4,17	28	-76	-50	24,5	-74,2	-49,4	30,8	30	-74	-50	23,8
4	149	4,52E-03	2,35	4,18	42	48	16	40,6	47,4	18,9	38,9	42	52	12	29,7
3	146	5,10E-03	2,29	3,91	20	-100	6	16,7	-101	3,96	57,5	12	-102	0	40,2
2	127	1,12E-02	1,95	4,03	2	-58	-24	2,84	-60,8	-29	19,7	4	-66	-38	15,9
1	105	2,94E-02	1,53	4,35	-30	22	2	-30	21,2	4,32	31,8	-32	20	8	21,5

Regressor: Pa

Cluster	Voxels	P	-log10(P)	Z-MAX	Z-MAX X	Z-MAX Y	Z-MAX Z	Z-COG X	Z-COG Y	Z-COG Z	COPE-	COPE-	COPE-	COPE-	COPE-
5	6009	0,00E+00	47,5	5,37	-16	-64	50	1,35	-59,5	53,1	245	10	-68	64	101
4	1157	1,81E-15	14,7	5,07	12	-92	-22	23,1	-80,7	-22,8	257	20	-90	-22	95,4
3	381	6,56E-07	6,18	5,03	36	-48	-54	36	-45	-50,1	81,7	36	-48	-54	49,9
2	159	1,85E-03	2,73	4,18	-28	-64	-28	-28,9	-64,3	-27,2	64,8	-26	-74	-22	47,9
1	132	5,97E-03	2,22	4,51	28	-4	52	27,3	-4,6	52,5	79,4	30	-6	58	56,1

Regressor: Pall[A > nA]

Cluster	Voxels	P	-log10(P)	Z-MAX	Z-MAX X	Z-MAX Y	Z-MAX Z	Z-COG X	Z-COG Y	Z-COG Z	COPE-	COPE-	COPE-	COPE-	COPE-
1	212	2,18E-04	3,66	3,88	30	-44	40	38,3	-42,4	44	100	48	-44	60	50,9

fMRI Decision Model 2

Regressor: Pall(tau_i=0)

Cluster	Voxels	P	-log10(P)	Z-MAX	Z-MAX X	Z-MAX Y	Z-MAX Z	Z-COG X	Z-COG Y	Z-COG Z	COPE-	COPE-	COPE-	COPE-	COPE-
2	875	3,02E-13	12,5	4,19	14	-66	58	-9,16	-62,8	55,9	125	-38	-56	58	63,9
1	188	3,72E-04	3,43	4,19	32	-44	42	36	-43,4	43,6	91,7	44	-48	60	44

Regressor: Pall(tau_i=1)

Cluster	Voxels	P	-log10(P)	Z-MAX	Z-MAX X	Z-MAX Y	Z-MAX Z	Z-COG X	Z-COG Y	Z-COG Z	COPE-	COPE-	COPE-	COPE-	COPE-
10	4389	1,69E-38	37,8	5,66	-54	-20	42	-27,9	-11,7	52,7	123	-46	-40	60	41
9	2880	7,49E-29	28,1	5,37	26	-48	-20	10,2	-74,1	-4,37	94,7	2	-88	0	38,8
8	697	1,17E-10	9,93	5,25	-16	-70	52	-18,1	-67,7	49	94,8	-16	-74	56	45,4
7	537	8,17E-09	8,09	5,87	-44	-66	-18	-43,5	-61,2	-18,4	74,6	-46	-70	-20	39,1
6	427	1,79E-07	6,75	4,9	-14	14	0	-18,1	10,7	0,875	25,9	-16	12	0	18,2
5	374	9,54E-07	6,02	4,68	14	16	2	18	11,9	2,72	24,9	20	10	-8	18
4	248	6,61E-05	4,18	4,14	-40	36	26	-38,7	37,1	23	41,1	-42	40	14	28
3	209	2,79E-04	3,55	4,61	52	-76	4	48,6	-79,6	-0,197	49,8	42	-88	-10	32
2	122	1,01E-02	2	4,82	-44	-2	6	-43	-0,926	8,51	33,7	-44	-2	8	22,1
1	89	4,80E-02	1,32	4,14	32	42	22	33,7	43,6	23,5	33,3	32	42	26	26,1

fMRI Feedback Model

Regressor: Win

Cluster	Voxels	P	-log10(P)	Z-MAX	Z-MAX X	Z-MAX Y	Z-MAX Z	Z-COG X	Z-COG Y	Z-COG Z	COPE-	COPE-	COPE-	COPE-	COPE-
10	4265	1,25E-37	36,9	5,5	-4	16	42	-24,5	-9,38	52,9	109	-46	-40	60	39,6
9	3327	9,47E-32	31	5,18	26	-46	-20	7,77	-76	-1,6	94,5	2	-88	0	37,3
8	482	5,96E-08	7,22	4,69	16	16	0	21,3	13,3	3,36	31,1	34	20	8	19,4
7	422	2,38E-07	6,62	5,05	-20	12	0	-17,6	12,1	1,49	28,8	-18	12	0	19,6
6	413	2,98E-07	6,53	5,62	-44	-66	-18	-43	-61,4	-16,4	67,3	-46	-70	-20	38,2
5	232	1,23E-04	3,91	4,1	-38	36	18	-38,6	37	21,9	42,4	-42	40	14	28,9
4	159	2,09E-03	2,68	4,5	52	-76	4	51,7	-75	3,9	43,9	48	-80	8	29,6
3	98	3,15E-02	1,5	4,58	-44	-2	6	-42,2	-1,39	9,29	33,2	-44	-2	8	22,2
2	93	4,01E-02	1,4	4,97	-8	-22	8	-11,1	-21,3	7,05	23,7	-8	-22	8	16,5
1	90	4,64E-02	1,33	4,45	-16	-70	52	-16,7	-69,7	50,4	68,1	-16	-72	54	44

Regressor: PE_Pall[A]

Cluster	Voxels	P	-log10(P)	Z-MAX	Z-MAX X	Z-MAX Y	Z-MAX Z	Z-COG X	Z-COG Y	Z-COG Z	COPE-	COPE-	COPE-	COPE-	COPE-
1	337	2,38E-06	5,62	4,3	-8	18	44	-3,15	18,6	43,6	21,9	0	22	46	

Regressor: PE_Pall[nA]

Cluster	Voxels	P	-log10(P)	Z-MAX	Z-MAX X	Z-MAX Y	Z-MAX Z	Z-COG X	Z-COG Y	Z-COG Z	COPE-	COPE-	COPE-	COPE-	COPE-
18	2597	1,20E-25	24,9	4,84	2	20	50	-4,71	14,4	49,9	99,6	-32	-4	70	34,8
17	1960	4,12E-21	20,4	4,38	-54	-22	40	-40,6	-38,9	49,5	126	-48	-40	60	42,6
16	1078	6,89E-14	13,2	5,35	-16	14	-2	-15,1	11,5	1,89	61,2	-6	2	10	25,4
15	967	7,40E-13	12,1	5,46	16	18	10	14,2	8,16	5,07	50,3	8	6	4	22,7
14	465	1,19E-07	6,92	3,99	26	-76	-16	15,8	-77,5	-12	67,4	2	-88	-4	40,4
13	327	8,52E-06	5,07	4,11	26	-54	-24	29,6	-51,9	-26,7	31,7	32	-52	-20	18,4

12	266	6,13E-05	4,21	3,99	-54	6	14	-51,5	9,51	21,5	64,3	-54	18	-4	32,6
11	262	7,00E-05	4,15	4,04	50	-68	0	51	-66,2	-2,78	44,3	52	-66	0	27,6
10	256	8,58E-05	4,07	4,25	-42	54	12	-40,4	49,7	10,2	53	-42	54	12	31,6
9	253	9,50E-05	4,02	4,11	36	-4	52	33,4	-4,22	56,5	75,1	32	0	66	36,2
8	224	2,60E-04	3,58	4,12	-14	-22	8	-10,2	-19,2	6,04	31,1	-2	-12	10	15,4
7	217	3,34E-04	3,48	4,29	58	14	4	54,6	10,5	14,8	48,7	60	14	2	27,9
6	134	8,21E-03	2,09	4,2	30	-38	42	35,9	-41,7	52,8	58,6	44	-40	62	33,5
5	133	8,57E-03	2,07	4,04	-32	-66	-24	-32,3	-57	-25,2	34,8	-34	-68	-22	22,6
4	116	1,78E-02	1,75	3,96	2	-58	-26	3,12	-61	-29,2	20,5	4	-68	-38	15,7
3	110	2,31E-02	1,64	4,07	30	-76	-50	21,4	-75,5	-49,4	28,9	30	-76	-50	23,3
2	107	2,64E-02	1,58	3,77	10	-104	4	16	-101	2,83	56,9	12	-102	0	39,5
1	95	4,56E-02	1,34	4,09	40	50	14	41,8	48,3	16,5	37,7	42	52	12	28,5

fMRI Feedback PPI seed IPS

Regressor: PPI [A > nA]

10	4265	1,25E-37	36,9	5,5	-4	16	42	-24,5	-9,38	52,9	109	-46	-40	60	39,6
9	3327	9,47E-32	31	5,18	26	-46	-20	7,77	-76	-1,6	94,5	2	-88	0	37,3
8	482	5,96E-08	7,22	4,69	16	16	0	21,3	13,3	3,36	31,1	34	20	8	19,4
7	422	2,38E-07	6,62	5,05	-20	12	0	-17,6	12,1	1,49	28,8	-18	12	0	19,6
6	413	2,98E-07	6,53	5,62	-44	-66	-18	-43	-61,4	-16,4	67,3	-46	-70	-20	38,2
5	232	1,23E-04	3,91	4,1	-38	36	18	-38,6	37	21,9	42,4	-42	40	14	28,9
4	159	2,09E-03	2,68	4,5	52	-76	4	51,7	-75	3,9	43,9	48	-80	8	29,6
3	98	3,15E-02	1,5	4,58	-44	-2	6	-42,2	-1,39	9,29	33,2	-44	-2	8	22,2
2	93	4,01E-02	1,4	4,97	-8	-22	8	-11,1	-21,3	7,05	23,7	-8	-22	8	16,5
1	90	4,64E-02	1,33	4,45	-16	-70	52	-16,7	-69,7	50,4	68,1	-16	-72	54	44
