# Supplementary material for: The parietal cortex has a causal role in ambiguity computations in humans
Source: PLoS Biol. 2024 Jan 10;22(1):e3002452. doi: 10.1371/journal.pbio.3002452 (PMC10824459; doi:10.1371/journal.pbio.3002452)
Supplement: S3 Table — Parameters of the different behavioral model adjusted for TMS experiments. The data underlying this table can be found at https://osf.io/zd3g7/. (PDF) [file pbio.3002452.s006.pdf]

Supplementary Table 3

TMS behavioral
Models

MODEL: Logit

	Lower95	Median	Upper95	Mean	SD	Mode	psrf	pMCMC	
beta0	-6,992	-5,433	-3,686	-5,433	0,855	-5,524	1,036	0,000	***
beta1 PV	5,898	7,817	9,776	7,834	0,991	7,843	1,026	0,000	***
beta2 Rw	1,602	3,499	5,441	3,512	0,984	3,449	1,016	0,000	***
beta3 PV*AM	-8,372	-5,347	-2,419	-5,335	1,536	-5,472	1,034	0,000	***
beta4 Rw*AM	-2,842	-0,142	2,908	-0,073	1,520	-0,189	1,023	0,468	
beta5 AM	-0,799	2,074	4,580	2,008	1,424	2,102	1,036	0,092	
beta0t TMS	-1,937	-0,345	1,227	-0,391	0,869	-0,285	1,086	0,351	
beta1t PV*TMS	-0,906	1,000	2,891	1,011	0,991	0,938	1,077	0,155	
beta2t Rw*TMS	-2,149	-0,586	1,040	-0,561	0,855	-0,787	1,060	0,269	
beta3t PV*AM*TMS	-7,412	-3,238	-0,137	-3,335	1,884	-2,958	1,061	0,027	*
beta4t Rw*AM*TMS	-3,105	0,406	4,203	0,325	1,976	0,157	1,035	0,434	
beta5t AM*TMS	-1,691	1,794	5,021	1,933	1,848	1,349	1,056	0,150	
deviance	2.783,320	2.829,205	2.878,410	2.830,358	24,445	2.824,596	1,000		

MODEL: tau_i

	Lower95	Median	Upper95	Mean	SD	Mode	psrf	pMCMC for TMS effects	
tau_i	0,342	0,718	1,163	0,737	0,213	0,697	1,003		
tau_i * TMS	0,070	0,484	0,885	0,497	0,206	0,481	1,003	0,008	**
alpha	0,109	3,476	12,624	4,630	3,930	1,733	1,001		
alpha*TMS	-4,434	-0,717	3,051	-0,706	1,942	-0,473	1,040	0,688	
gamma	1,948	2,947	4,007	2,972	0,535	2,871	1,002		
gamma * TMS	-0,233	0,043	0,495	0,061	0,167	0,032	1,005	0,660	
beta_0	-0,213	-0,036	0,124	-0,037	0,086	-0,045	1,000		
beta_0* TMS	-0,249	-0,044	0,154	-0,043	0,105	-0,055	1,000	0,670	
beta_1	3,871	8,255	12,741	8,274	2,284	8,203	1,002		
beta_1 * TMS	-0,318	2,728	5,461	2,745	1,430	2,696	1,007	0,054	
deviance	2.866,900	2.902,355	2.935,600	2.902,180	17,902	2.902,536	1,003		

MODEL: tau_i + tau_b

	Lower95	Median	Upper95	Mean	SD	Mode	psrf	pMCMC for TMS effects	
tau_i	0,249	0,583	0,979	0,595	0,186	0,542	1,001		
tau_i * TMS	0,141	0,491	0,864	0,507	0,187	0,468	1,000	0,001	***
tau_b	0,292	0,400	0,503	0,400	0,053	0,405	1,003		
tau_b* TMS	-0,020	0,069	0,157	0,071	0,045	0,067	1,006	0,105	
alpha	0,816	1,002	1,193	1,000	0,117	1,097	1,000		
alpha*TMS	1,932	2,974	4,131	3,008	0,563	2,869	1,001	0,945	
gamma	-0,497	2,461	5,016	2,527	1,375	2,442	1,006		
gamma * TMS	-0,381	-0,127	0,126	-0,125	0,128	-0,126	1,001	0,720	
beta_0	-0,347	0,046	0,464	0,054	0,201	0,035	1,007		
beta_0* TMS	-1,738	-0,085	1,876	-0,036	1,001	-0,011	1,005	0,314	
beta_1	3,563	8,024	12,672	8,069	2,318	7,716	1,002		
beta_1 * TMS	2.855,570	2.891,910	2.929,850	2.892,347	19,509	2.891,273	1,002	0,063	
deviance	-0,113	0,093	0,302	0,093	0,106	0,081	1,001		

MODEL: tau_i + tau_b

	Lower95	Median	Upper95	Mean	SD	Mode	psrf	pMCMC for TMS effects	
tau_i	0,347	0,731	1,145	0,751	0,208	0,706	1,021		
tau_i * TMS	0,017	0,510	1,033	0,516	0,256	0,510	1,008	0,009	**
alpha	0,108	1,023	1,898	1,031	0,542	1,118	1,000		
alpha*TMS	-1,816	-0,041	1,841	-0,009	1,045	-0,128	1,041	0,976	
gamma	1,659	2,677	3,743	2,703	0,528	2,611	1,102		
gamma * TMS	-0,469	-0,013	0,430	-0,005	0,222	-0,027	1,032	1,000	
beta_0	-0,203	-0,020	0,156	-0,019	0,092	-0,022	1,006		
beta_0* TMS	-0,291	-0,054	0,148	-0,057	0,112	-0,061	1,006	0,597	
beta_1	5,404	9,172	13,568	9,198	2,064	9,343	1,040		
beta_1 * TMS	0,297	2,463	4,933	2,483	1,169	2,423	1,014	0,037	*
Wr	-0,483	-0,164	0,162	-0,166	0,161	-0,163	1,003		
Wr*TMS	-0,398	-0,029	0,234	-0,039	0,160	-0,003	1,012	0,846	
deviance	2.776,530	2.813,050	2.854,830	2.814,659	20,656	2.810,781	1,041		
