# Supplementary material for: The parietal cortex has a causal role in ambiguity computations in humans
Source: PLoS Biol. 2024 Jan 10;22(1):e3002452. doi: 10.1371/journal.pbio.3002452 (PMC10824459; doi:10.1371/journal.pbio.3002452)
Supplement: Attachment — Submitted filename: Answers2Reviewers_1.docx [file pbio.3002452.s007.docx]

We would like to thank the editor for allowing us to submit a revised version of our manuscript and the reviewers for their valuable comments. Thanks to the feedback we received, we have made several changes to the manuscript that have enhanced the robustness of our findings, as follows.

1. We have added an analysis demonstrating the influence of prediction error on the perception of ambiguity in the subsequent trial. This contributes to the rationale behind the design of TMS before feedback and the study of prediction error during the feedback period both in fMRI and EEG [new version of Figure 2D].
2. A new analysis, which correlates BOLD activity with behavior, supports the hypothesis regarding the direction of the TMS effect.
3. An additional analysis during the decision-making period in the EEG-TMS experiment provides further evidence of the mechanism through which TMS influences behavior. Thus, it appears that the disruption of the fronto-parietal network may underlie the perception of uncertainty during decision-making under ambiguity.

In the following paragraphs, we provide a detailed account of the new analyses and manuscript revisions, addressing each reviewer's comments individually.

REVIEWS:

Reviewer #1: In this work, the authors demonstrated that the parietal cortex plays a causal role in computing ambiguous probabilities, using a very nice design that embrace a robust and multi-method perspective (behavioural modeling, fMRI, TMS, EEG). They found that participants elevated the uncertainty associated to their decisions and that parietal activity correlated with the underestimation of the uncertainty during decision-making. Moreover, disruption of parietal activity by means of TMS influences the assignment of ambiguous probabilities, increased the uncertainty associated to decision-making. In addition, they found that the midcingulate cortex encodes prediction errors and increases its connectivity with the parietal cortex during outcome processing.

The work is well done and provides an exciting contribution to the topic. In addition, they followed a sequential approach in which the results of one neuroscientific technique (e.g., fMRI) guided the investigation that was carried out with another set of techniques (TMS-EEG), enhancing the validity of the approach and robustness of the findings.

In what follows I provide some comment which I hope can support the authors in further improving this already excellent work.

Comments:

R.1.1)	In the results section, you stated that "we first aimed to examine the intra-block cumulative effect of trial-by-trial TMS on behavior. To do this, we analyzed the behavioral effects in the last 20 trials of each 40-trial block of the same TMS stimulation."

I feel that it might be helpful to further clarify why you expect a cumulative effects of the TMS protocol applied.

A.1.1)	We appreciate the reviewer's comments. The assessment of the cumulative effect of stimulation was initially conceived concerning the changes induced by the feedback, so we decided to introduce the TMS stimulation before the feedback. Indeed, in light of the new results in the new version of the manuscript, we now provide further evidence supporting this rationale (see A.3.3, new Figure 2D, and results on Page 7 Paragraph 2). We now show that the preceding feedback influences τ_i_ parameters for the subsequent trial, indicating that following unexpected feedback (i.e., with greater ppe), participants made adjustments in their subsequent decisions, reducing the perceived uncertainty. Thus, and as detailed in our new analysis, we observed that the probability prediction error of the preceding trial influences the τi parameter. Given that TMS stimulation introduces a consistent perturbation to the prediction error signal within a block of trials, it is conceivable that this perturbation might accumulate over subsequent trials. It is possible to propose that this sustained influence could lead to a discernible effect that becomes more evident as trials progress. For this reason, we considered the analysis of the last 20 trials. We added this rationale to the main text (Page 10, Paragraph 2).

Figure 2. Behavioral Results. A. Rate of choice where individuals preferred the highest probability per conditions. B. Rate of choice where subjects preferred the highest probability per degree of ambiguity. Black dots represent the rate per individual. Color rectangles indicate the number of individual decisions; red represents the maximum, and light gray represents the minimum account. The blue line represents the linear regression, and the gray area is the standard error. C Model fitting comparison using DIC, red, and LOOIC, green. D Posterior distribution of the key parameters for each model. Black dots represent the mean of the distribution, and black lines the 95% high-density intervals. The colored areas represent the complete posterior distribution. E Correlation between decision shift (difference between the rate of choices that the subject prefers the highest probability between condition, ambiguity less no-ambiguity conditions), and i parameters. Red dots represent each subject. The solid blue line represents the linear regression, the dotted blue line the LOESS regression, and the gray area represents the standard error. * indicates p<0.05, ** p<0.01,***p<0.001. See also Supplementary Figure 1 and Supplementary Table 1.

R.1.2)	Connected to the previous point, you reported in the text that "in the TMS-EEG experimental session, participants completed 6 runs of TMS stimulations, consisting of 2 runs of 40 trials with TMS interference on the PPC, two runs of 40 trials with TMS interference at the IPS, and two runs of 40 trials with TMS interference at the vertex, as an active control condition." Considering that you have analyzed the behavioral effects in the last 20 trials of each 40-trial block of the same TMS stimulation, it means that, you have considered the contribution of 40 trials, in turn divided in non-ambiguous and ambiguous trials, for each TMS condition? I would like to have more clarification on this, considering that this number might be too small to have a stable measure of the effect of TMS on behavioral indices.

A.1.2)	We thank the reviewer for this valuable comment. As the reviewer notices, the TMS effect analyzed in our study consists of 40 trials (20 ambiguity and 20 no ambiguity) for each subject for TMS stimulation condition (20+20 IPS, 20+20 PPC, 20+20 sham). We are aware that having more trials could be useful to obtain a stable behavioral effect. The rationale for our methodological decision was to obtain a larger number of trials without losing the power of within-subject analysis. On the contrary, increasing the number of trials would have considerably extended the required experiment duration, compelling us to either split the TMS experiment into two sessions or allocate it across two participant groups. This would have undermined the robustness of the statistical analysis approach, demanding a shift from within-subject to between-subject analysis. Hence, our primary analysis assesses the combined effect of the two parietal TMS conditions, leading to a more accurate statistical estimation. However, in an effort to account for potential distinctions across brain regions, we conducted separate independent analyses for the IPS and PPC. The results revealed no statistically significant differences (difference TMSppc - TMSips mean = 0.28, HDI = [-0.29 0.97], p_MCMC_=0.35; see A.2.2). Regarding cognitive effects; it is difficult to define an optimal point to elicit behavioral effects because the measure depends on several factors such as the task and context of stimulation. However, our experimental design considered more TMS pulses than the minimum threshold required to elicit reliable physiological effects (Chang et al., 2016; Brownstein et al., 2018, suggest an optimal range of 18 to 25 pulses).

We now include these methodological considerations in the Methods section of the revised version of the paper as follows: “The rationale for this block design was to obtain a balance that maximizes the number of trials per condition while maintaining a single TMS-EEG session for each subject [80,81]. This approach allowed us to enhance our statistical power through within-subject analysis.” (Page 23, Paragraph 1).

References:

Chang, W. H. *et al.* Optimal number of pulses as outcome measures of neuronavigated transcranial magnetic stimulation. *Clin. Neurophysiol.* **127**, 2892–2897 (2016).

Brownstein, C. G. *et al.* An optimal protocol for measurement of corticospinal excitability, short intracortical inhibition and intracortical facilitation in the rectus femoris. *J. Neurol. Sci.* **394**, 45–56 (2018).

R.1.3)	If I understand correctly, the activity of the right parietal regions is correlated with a tendency to calculate options as more certain than they actually are, and the disruption of their activity by TMS leads participants to change their decisional strategy, behaving as if they perceive greater uncertainty in decision-making. These findings resemble recent research (e.g., Tarasi et al., Prog. Neurobiology 2022) that showed that right parietal areas are involved in the modulation of decision-making strategies. This same research, however, demonstrates that this strategical adjustment is not linked to a concurrent increase in objective performance. Would it be possible to demonstrate whether TMS stimulation of the parietal area may have influenced the participants' objective performance or whether it modulated only the decision-making strategy? For example, is it possible to show whether the "final" reward obtained by the participants differs significantly according to

the TMS condition or remains stable between the tested conditions?

A.1.3) We appreciate the reviewer's comment. To address this question, we analyzed whether participants' "final" reward differed significantly according to the TMS condition or remained stable between the tested conditions. Our results indicate no significant differences in the final reward obtained by participants across the different TMS conditions. This suggests that the TMS stimulation of the parietal area primarily influenced the decision-making strategy rather than directly impacting objective performance. Furthermore, we incorporate simulation results that provide additional insights into the effects of these strategic changes (see also A.3.1).

We now include these results in the new version of the manuscript (Page 11, Paragraph 1).

Reward:

Fixed effects:

Estimate Std. Error z value Pr(>|z|)

(Intercept)	-2.5211	0.1865 -13.517	<2e-16 ***

TMS	-0.1214	0.2369 -0.513	0.6083

Ambiguity	0.5490	0.2461	2.231	0.0257 *

TMS: Ambiguity	0.1588	0.3062	0.519	0.6041

Positive feedback (win):

Fixed effects:

Estimate Std. Error z value Pr(>|z|)

(Intercept)	0.708793	0.168422	4.208 2.57e-05 ***

TMS	0.037441	0.160279	0.234 0.81530

Ambiguity	-0.587058	0.187346 -3.134 0.00173 **

TMS:Ambiguity	-0.003725	0.196861 -0.019 0.98490

Interestingly, these results align with the paper discussed by the reviewer, in terms of the differences in strategies and patterns of the brain activity identified but not in the objective performance, understood as gains and reinforcement obtained. We now added this interpretation in the Discussion Section (Page 15, Paragraph 2).

R.1.4)	In the paper, the activity from the parietal to the frontal is mainly treated since the former would provide outcome predictions made through ambiguity computation needed to calculate the prediction error. Is it possible to discuss whether there could also be a flow that travels in the opposite direction (from frontal-to-parietal) that "adjusts" the ambiguity computation after a commission of an error?

A.1.4) We thank the reviewer for this valuable comment. Certainly, due to the observed contribution of frontal activity in both fMRI analysis and EEG source analysis, it would be possible to argue that frontal-parietal communication could occur bidirectionally. Indeed, our new results showed that after feedback with a high PPE, the participants adjusted their τ_i_ by diminishing it in the subsequent trial (See also A.1.1; A.3.3). We added this interpretation in the Discussion Section (Page 15, paragraph 2).

R.1.5)	Would it be possible to demonstrate whether there are any oscillatory signatures of the role of the parietal regions when computing ambiguous probabilities?

A.1.5) We appreciate the reviewer for this comment. To address this topic, we conducted the following analysis. Since we did not have a specific hypothesis regarding which oscillatory activity and electrodes might reflect modulation related to parietal cortices in ambiguity, we performed an exploratory whole-scalp analysis. Specifically, in the time window preceding the subject's button-press decision, we found a significant modulation in theta and alpha frequency ranges. Interestingly, this activity correlated with the probability of the chosen option during ambiguity (P_all_ [A > nA]). When analyzing the effect of TMS as a regressor, we found that this activity was attenuated, resulting in a significant negative interaction. Strikingly, this modulation originated from cortical sources in regions near the FEF, in a cluster similar to that found in fMRI analysis. Specifically, this region correlated with the degree of ambiguity in fMRI. We included these results in the new Figure 6 in the revised version of the manuscript. (Page 13, Paragraphs 2 and 3.)

|  | **Figure 6. Oscillatory brain activity in frontal electrodes associated with the probability of the chosen option during the decision period. A.** The time-frequency chart illustrates differences in frontal electrodes between the Ambiguity and No-Ambiguity conditions in the correlation between oscillatory power and the probability of the chosen option (Pall[A > nA]). **B.** The joint effect of two models, one for Vertex TMS stimulation and the other for the difference between Vertex TMS and parietal TMS stimulation (TMS effect). **C.** A separate analysis of theta activity for TMS stimulation in the IPS and PPC. **A-B.** The highlighted areas indicate time-frequency epochs with significant modulation, determined using a cluster-based permutation test (CTD: p < 0.05, Wilcoxon test). **A-C.** Scalp topologies display oscillatory activity in theta frequency range. Source estimation for theta activity is provided for Vertex and the TMS effect. Magenta lines on the cortex represent areas where BOLD activity correlates with Pa in the fMRI experiment (see Figure 3). All source results are presented on a high-resolution mesh for visualization purposes only. |
| --- | --- |

Reviewer #2: The behavioural results that the authors demonstrated are convincing. The shift in behavior during ambiguous decisions is associated with a selective decrease in probability weighting.

The model that the authors apply is appropriate. It is great that the robustness of the model was confirmed by an independent data set.

fMRI experiments and analyses were carefully conducted and the reviewer was convinced by the authors' conclusion that activity in the bilateral IPS and PPC reflects the degree of ambiguity.

R.2.1) Causal evidence by the TMS experiment is also convincing but it is a bit uncertain for the reviewer about the difference in causal impact by stimulation between IPS and PPC.

The authors state that they merged data from IPS and PPC stimulation as there are no differences in their effects on behavior. But the reviewer thinks that it is worth demonstrating the data with IPS stimulation and PPC stimulation separately.

A.2.1) We appreciate the reviewer's comment. As mentioned in our response to R1 (A.1.2), ensuring an adequate number of trials is crucial for obtaining robust results in our study. To address this concern, we have conducted a revised analysis approach.

For the raw behavioral markers, we conducted mixed-effects general models, separating the TMS stimulation conditions. These analyses revealed that both stimulations had a significant impact on behavior, and we did not find differences between them.

Ambiguity condition.

Ambiguity and IPS interaction, beta = -0.32, s.e. = 015, z-value = -2.0, p = 0.03;

Ambiguity and PPC interaction, beta = -0.39, s.e. = 0.15, z-value = -2.6, p = 0.009;

Correlation with the Ambiguity degree:

Ambiguity degree and IPS interaction, beta = -0.51, s.e. = 0.24, z-value = -2.1, p = 0.03;

Ambiguity degree and PPC interaction, beta = -0.59, s.e. = 0.23, z-value = -2.4, p = 0.01;

(Page 11, Paragraph 1)

For cognitive modeling, we have utilized a unified model to investigate the impact of τ_i_ across different stimulations rather than conducting separate analyses for each stimulation. This approach allows us to maintain a sufficient number of trials for accurately determining all other parameters of the model. By employing this methodology, we have generated new findings that highlight an increase in τ_i_ for both TMS stimulations. These observed effects were statistically significant. Importantly, our analysis did not identify any significant differences between the two stimulations in terms of their effects on τ_i_.

mean	[DHI]	(p_MCMC)

Tau_i_ppc: 0.62	[0.06 1.27]	0.012

Tau_i_ips	0.33 [0.01 0.72]	0.045

Tau_i_dif(ppc-ips)	0.28	[-0.29 0.97]	0.35

(Page 12, Paragraph 1)

R.2.2) Especially it is interesting if the authors show EEG data with TMS stimulation separately for the IPS stimulation condition and PPC stimulation condition. How was the effect of EEG signals around PPC caused by IPS stimulation? How about EEG signal changes around IPS caused by PPC stimulation? It will help the authors to dissociate the roles of IPS and PPC for value-based decision making with consideration of ambiguity.

A.2.2) We appreciate this reviewer’s comment. As we pointed out in response A1.2, the number of trials in our experiment does not allow us to draw strong inferences when separating the TMS conditions in the IPS and PPC. However, following the reviewer's suggestions, we added separate analyses of each parietal stimulation in the EEG signal to our joint analysis.

For feedback activity, “The separate analysis of the IPS and PPC TMS stimulations revealed that both modulated the MCC delta activity to varying degrees (Figure 5C). When comparing these TMS stimulations, a modulation effect was observed near the PPC stimulation site (see Figure 5C).” Page 12 Paragraph 3, see new Figure 5

For the new analysis of decision-related activity (See A.1.5., and new Figure 6), “The separate analysis of the IPS and PPC TMS stimulations revealed that only the IPS exhibited significant modulation in the frontal eye-field region, with no differences between the two stimulations (Figure 6C).” (Page 13, Paragraphs 2 and 3, and new Figure 6 in A.1.5 and new Figure 5.)

|  | **Figure 5. Oscillatory brain activity in frontal electrodes associated with unsigned prediction error during feedback.** **A.** Time-frequency chart in frontal electrodes for the correlation between oscillatory power and unsigned prediction error given by τ_i_=0 model (uPE-P_all(τi=0)_), τ_i_=1 model (uPE-P_all(τi=1)_), and de join effect of two models for both Vertex TMS stimulation and the difference between vertex TMS and parietal TMS stimulation (TMS effect). The highlighted areas indicate time-frequency epochs showing significant modulation (without time-frequency a priori, cluster-based permutation test, CTD: p <0.05 Wilcoxon test). Scalp topographies show oscillatory activity in the delta range. **B.** Source estimation for delta activity correlated with unsigned prediction error given by τ_i_=0 model (uPE-P_all(τi=0)_) for Vertex and TMS effect. Sources that survive multiple comparison corrections are shown (FDR q <0.05). The highlighted areas (green and red lines in the inserts) represent the coincident areas for EEG source estimation and BOLD activity for the fMRI experiment. All source results are shown in a high-resolution mesh only for visualization purposes. **C.** A separate analysis of delta activity for TMS stimulation in the IPS and PPC, and the differences between them. |
| --- | --- |

R.2.3) The authors discuss that correlation between frontal delta and theta activity and prediction errors in uncertain situations is known in previous studies. In the present study they clearly demonstrate a reduction in delta activity in the MCC during feedback caused by parietal disruption. That is one of the most remarkable findings of the study. But the reviewer supposes that there is a possibility that MCC plays a causally essential role in the computation of ambiguous information rather than the parietal cortex. The reviewer proposes that the authors should demonstrate the predictability of behavioural performance by the BOLD signal in PPC, IPS and MCC, separately, during the fMRI experiment and compare them. This analysis will strengthen the authors' point that parietal cortex plays essential role rather than MCC.

A.2.3) We appreciate the reviewer's comment. To address this, in the fMRI analyses, we extracted the BOLD signal during the decision-making period from two ROIs identified by (i) the analysis of τ_i_ =0 [parietal ROI], as well as informed by (ii) the functional connectivity result [MCC ROI]. Behavioral changes are exclusively predicted by activity in the parietal ROI, with no significant influence observed from MCC activity.

We added this in the result section:

“To assess the influence of these identified areas on behavior, we examined whether BOLD activity in the parietal region could predict behavioral shifts between non-ambiguous and ambiguous conditions, as observed in the behavioral analysis (Figure 2A). We employed mixed logit models to predict whether subjects preferred the more probable or rewarded option on a trial-by-trial basis. We extracted the BOLD signal during the decision-making period from a parietal region of interest (ROI), identified by the τi=0 model (fMRI-Model 2) and an MCC ROI based on uPE-P_all_ [A] analysis (see Figure 3). Consistent with behavioral analysis, participants in the ambiguity condition shifted their preference toward the more rewarded option (ambiguity regressor: beta=-1.08, s.e.=0.1, t=-8.8, p < 1e-10). Parietal activity significantly attenuated this effect (Parietal-ambiguity interaction: beta=0.27, s.e.=0.12, t=2.2, p=0.02, d.f=1436) in line with the interpretation that the parietal cortex reflects decisions made with greater certainty (see Figure 3A). No impact was observed during the no-ambiguity condition (Parietal regressor: beta=-0.15, s.e.=0.1, t=-1.5, p=0.12;). No significant effects were found for the MCC ROI (MCC-Ambiguity interaction: beta=0.15, s.e.=0.11, t=1.2, p=0.19, d.f=1436).” Page 10 Paragraph 1

R.2.4) Please double-check figure legend. Some information, especially statistical values are invisible.

A.2.4) We appreciate the reviewer for this observation. In the new version of the manuscript, we conducted a double-check of all figure captions.

Overall, the manuscript seems to be persuasive and well-organised. The reviewer thinks that it reaches the criterion to publish from PLOS Biology if they sort out the aforementioned issue.

Reviewer #3: The manuscript investigates the role of the parietal cortex in decision-making involving ambiguity, combing computational modeling, fMRI and TMS-EEG technique. In particular, the paper proposes an internal process by which ambiguity is converted into a subjective probability distribution in service of decision-making and tested this proposal using neuroscience tools. Their results point to a role of the parietal cortex which seems to be related to ambiguity processing in a number of different ways. Overall, the research question is important and the paper addresses the question using both correlational and causal tools. However, the writing of the paper should be significantly improved: some writing are quite sloppy, some important technique details are missing, and some figures are not cited in the main text (e.g., Fig 4B). Also, there are a few major questions need to be addressed.

A.3.1) We appreciate the reviewer's comment. We improve the writing of the new version of the manuscripts.

R.3.2) computational model. It would be helpful to include a couple of model simulation to clearly demonstrate the intuition. The paper should provide rationale and discussion of the proposed model, especially in light of the existing literature. Whether and how can the model address well-established findings such as ambiguity aversion? As far as I understand, ambiguity aversion should be closely related to the value of tau_i. But according to the paper, the value estimates of this parameter seem to vary quite a bit across two behavioral datasets, raising questions on the validity of the model.

A.3.2) We appreciate the reviewer's comment on our article. In response to this comment, we added the rationale of the proposed model based on previous formulations for studying ambiguity aversion and attitudes toward ambiguity. In a paragraph in the Results Section, which we have copied below, we showed this.

“Next, we investigated the potential cognitive processes underlying the observed behavioral effects. Prior studies examining the choices between ambiguous and risky or safe options have employed two major modeling approaches. The first approach involves determining the degree of ambiguity aversion by adjusting a parameter that discounts the objective probability, assuming no decision bias (see [28,29] for detailed formulations). This discount parameter can take on positive values, indicating ambiguity aversion, or negative values, signifying ambiguity-seeking tendencies. This discount parameter, however, may not accurately capture the perceived degree of ambiguity or uncertainty in the decision-making process. The second approach involves parameters associated with attitudes toward ambiguity. These parameters capture an individual's optimism or pessimism regarding participants' beliefs about ambiguous information, e.g., [30]. This parameter can be interpreted as a bias parameter in assigning ambiguous information, assuming a constant perception of uncertainty. Based on the preceding formulations, we proposed a cognitive computation to distinguish between the perceived degree of ambiguity and potential probability assignment biases in the decision-making process.” (Page 5 Paragraph 4.)

Additionally, we have added a section on simulations where all model parameters were held constant, with the sole variation being the τ_i_ parameter. These simulations enable us to provide specific insights into model behavior and interpretations concerning how parameters vary under different experimental conditions.

The new text is the following:

“**Model simulations**

We utilized the parameter readout from the τ_i_ model to simulate data using various generative τ_i_ values, replicating the same choice scenarios as those encountered by experimental participants. Our findings demonstrate the successful recovery of the generative τ_i_ values (see Supplementary Figure 2). Furthermore, we assessed how other variables vary as a function of τ_i_ (Supplementary Figure 3). Higher τ_i_ values were associated with a shift in preference towards more rewarding options, accompanied by an increase in the mean reward obtained. Consequently, this shift led to a reduction in the mean rate of winning, resulting in a greater mean reward prediction error. The probability prediction error exhibited a peak near τ_i_ around 1, declining for values both lower and higher than this threshold. Therefore, the influence of PPE in the τi of the next decision, as illustrated in Figure 3D, appears to be an effective means to diminish prediction errors in subsequent decision-making. ” (Page 7, Paragraph 3.)

|  | **Supplementary Figure 3.** Distribution of model variables derived from simulations as a function of the τ_i_ parameter. **A.** Mean reward obtained in a game as a function of the τ_i_ parameter. **B.** Mean rate of positive feedback or winning obtained in a game as a function of the τ_i_ parameter. **C.** Mean probability prediction error (PPE) obtained in a game as a function of the τ_i_ parameter. **D.** Mean reward prediction error (RPE) obtained in a game as a function of the τ_i_ parameter. **E.** Mean rate of choosing the more probable option in a game as a function of the τ_i_ parameter. |
| --- | --- |

Finally, the variation in the τ_i_ parameter across different data sets is expected, as all three samples involve experiments with different contexts. In the second data set, experiment modifications were employed to assess biases in decision-making. Furthermore, to enhance the validity of our formulation, we compared it with models formulated for ambiguity aversion using a discount parameter. Our model exhibits a better fit than this discount-parameter-based model as described in the Results Section in the new version of the manuscript as follows.

“Finally, we compared the adjustments made to the τ_i_ model with a discount parameter model (ambiguity aversions [28]). Our model demonstrated superior performance to this model (DIC difference = 19.5 and LOOIC difference = 50.2).” (Page 7, Paragraph 2.)

R.3.3) Another issue is that the model only considers the decision-making process. In neural data, however, the feedback (and prediction error) was proposed to play an important role. It would be helpful to formally model how feedback would influence subsequent decisions, and provide a quantitative framework for understanding the neural data.

A.3.3) We appreciate the reviewer's comment. In the new version of the manuscript, we have analyzed how the prediction errors in a trial impact the decision-making process in the subsequent trial. This approach allows us to assess explicitly how one trial influences the next. Behavioral analysis reveals that more prediction errors in a trial influence subsequent decision-making during ambiguity, reducing the value of the τ_i_ parameter. Interestingly, this serves as a means to reduce prediction errors in the following trials, as indicated by the results of our simulated analysis (see also A.1.1, A.3.2).

We add a new part in Figure 2 and this text in the results section:

“We conducted two controlled analyses. One of these analyses incorporated a parameter (Wr, see Methods) to address potential biases towards options with greater rewards during ambiguous situations. The other analysis included two parameters (β valency:τ_i_, β ppe:τ_i_ that aimed to explore the possible influence of feedback valency (win or no win) and probability prediction error on the τi parameter in the subsequent trial. Although the results showed no significant adjustment improvement for the control models, these reveal two behavioral features. First, the Wr parameter was no different from zero, indicating no evidence of ambiguity biasing decisions toward options with greater rewards (Wr mean = 0.5; HDI = [-0.05 1.3]; pMCMC=0.08; Figure 2D). Secondly, the value of β ppe:τi was significantly less than zero, indicating that following unexpected feedback (e.i., with greater ppe), participants made adjustments in their subsequent decisions, leading to a reduction in the perceived uncertainty (β ppe:τ_i_ mean = -0.87; HDI = [-1.5 -0.21]; pMCMC<0.001; Figure 2B). Nonetheless, feedback valency did not influence τi parameter (β valency:τ_i_ mean = -0.1; HDI = [-0.31 0.09]; pMCMC=0.3).” (Page 7, Paragraph 2.)

R.3.4. fMRI. I personally find that the writing of the fMRI part was difficult to follow. The paper needs to provide a better explanation of why a specific analysis was performed, and whether there are confounding factors that need to considered. For example, to which extent does P_all ( tau_i = 0) is correlated with P_all ( tau_i = 1)? whether the finding of the left parietal cortex can be explained by the correlation between the two?

A.3.4) We appreciate the reviewer's comments. In the revised version of our manuscript, we explain the objectives for each analysis before describing the results in the fMRI section. Additionally, recognizing that many regressors exhibit some degree of collinearity that can potentially confound the results, we have orthogonalized certain regressors in both models. For example, we have orthogonalized Pall to Pa. In the second model, the activity of Pall calculated with tau=1 and tau=0 has been orthogonalized with each other. Furthermore, in response to the suggestions made by Reviewer 2 (see A.2.3), we conducted an analysis correlating BOLD activity with subsequent behaviors, which revealed a correlation between parietal activity and a reduction in the behavioral effect of ambiguity. This is in line with the interpretation that parietal activity is involved in a computation that leads to perceiving the decision with less uncertainty. As a result, the primary fMRI findings exhibit a degree of robustness across various analyses. This serves to rule out the possibility that the main fMRI results are due to confounding effects.

R.3.5) TMS. The overall design was quite confusing to me. why focus on the feedback period? Whether and how should feedback influence choices? Whether the trials are related to one another (i.e., what needs to be learned from previous decision)? Do subjects actually show any learning effect in the behavioral experiment? For the TMS behavioral results, only mean value and non-parametric p value was reported. The paper needs to include other important statistics such as confidence interval, SEM or quantile, etc.

A.3.5) We thank the reviewer for these comments. In the revised version of the manuscript, we have explicitly explained the rationale behind using TMS before feedback. We have incorporated a new analysis demonstrating how a trial's prediction error significantly impacts the subsequent decision's decision-making process. See details in A.3.2. Furthermore, we have included the missing results for the mean and confidence interval in the behavioral TMS results.
