# Supplementary material for: The parietal cortex has a causal role in ambiguity computations in humans
Source: PLoS Biol. 2024 Jan 10;22(1):e3002452. doi: 10.1371/journal.pbio.3002452 (PMC10824459; doi:10.1371/journal.pbio.3002452)
Supplement: Attachment — Submitted filename: Reviewer responses.docx [file pbio.3002452.s008.docx]

Editor’s Comments

1) TITLE: We think the title might flow better if slightly re-arranged. We will ultimately defer to you, but suggest you consider changing it to "The parietal cortex has a causal role in ambiguity computations in humans"

We thank the editor for this valuable comment. We changed the title following the editor's suggestion in the revised version of the manuscript.

2) ETHICS STATEMENT: Please update the ethics statement in your methods section, to include the approval number for the protocol approved by the Ethics Committee of the Universidad del Desarrollo, Chile. Please also update this to indicate whether consent was written, or not.

We thank the editor for these comments. We will now provide additional details regarding the information that the informed consent was in writing and the ethical approval number (Folio 2020-67). [Page: 18, Paragraph: 1].

3) DATA: Thank you for providing the underlying data for your study as a deposition to OSF. Can you please add a brief sentence to each relevant figure legend (including supplemental) referencing this data? For example, you can add the sentence "the data underlying this figure can be found at [https://osf.io/zd3g7/"](https://osf.io/zd3g7/%22)

We have incorporated the suggested statement for referencing the OSF repository in both the figure and table captions.

.

Reviewers' Comments

Reviewer #1: The authors have adequately addressed all the concerns to full satisfaction. I have no further comments.

We appreciate Reviewer 1 for providing valuable feedback on our work.

Reviewer #2, Kentaro Miyamoto (note, reviewer 2 has signed this review): The authors fully respond to my comments with additional data analyses. Now, I'm convinced that the causal effects on behaviour by PPC and IPS are comparable. However, we have to reserve that a collection of the larger number of trials for each session is required to draw a firm conclusion about the distinction between the roles of PPC and IPS.

We thank the reviewer for this valuable comment. We agree with the reviewer that it is not possible to conclude that the PPC and IPS have exactly the same computational role in the ambiguity computation. We now include this issue in the Discussion section, and we argue that in order nceryo detect possible differences between the PPC and IPS further experiments are necessary. [Page: 15, Paragraph: 2].

For point 3, it is remarkable that BOLD signal in the parietal ROIs could predict behavioural performance, whereas that in MCC ROI. Direct statistical comparisons of the predictabilities between parietal and MCC ROIs will strengthen the author's point.

We thank the reviewer for this comment. We conducted a statistical test to assess the models’ predictive capabilities of the model using measurement adjustments. For parietal models, the inclusion of the regressor of interest (the interaction between parietal bold signal and ambiguity) resulted in a significant improvement in the fit (Chi-squared = 4.9, p-value = 0.02), while for the MCC model, there was no such improvement (Chi-squared = 1.6, p-value = 0.1947). However, we did not find evidence of a fit difference between the parietal and MCC models (Chi-squared = 1ared = 1.9, p-value = 0.16). [Page: 10, Paragraph: 1].

Reviewer #3: The revision has addressed my concerns. I have no further questions.

We thank to Reviewer 3 for all his/her valuable comments.
